# Adaptations in Reactive Balance Strategies in Healthy Older Adults After a 3-Week Perturbation Training Program and After a 12-Week Resistance Training Program

**DOI:** 10.3389/fspor.2021.714555

**Published:** 2021-10-20

**Authors:** Tom Van Wouwe, Maarten Afschrift, Sebastiaan Dalle, Evelien Van Roie, Katrien Koppo, Friedl De Groote

**Affiliations:** ^1^Human Movement Biomechanics Research Group, Department of Movement Sciences, KU Leuven, Leuven, Belgium; ^2^Department of Mechanical Engineering, Robotics Core Lab of Flanders Make, KU Leuven, Leuven, Belgium; ^3^Department of Human Movement Sciences, Vrije Universiteit, Amsterdam, Netherlands; ^4^Exercise Physiology Research Group, Department of Movement Sciences, KU Leuven, Leuven, Belgium; ^5^Physical Activity, Sport & Health Research Group, Department of Movement Sciences, KU Leuven, Leuven, Belgium

**Keywords:** reactive balance, perturbation-based balance training, resistance training, ankle strategy, hip strategy, older adults, stepping threshold, fall prevention

## Abstract

Both resistance training (RT) and perturbation-based training (PBT) have been proposed and applied as interventions to improve reactive balance performance in older adults. PBT is a promising approach but the adaptations in underlying balance-correcting mechanisms through which PBT improves reactive balance performance are not well-understood. Besides it is unclear whether PBT induces adaptations that generalize to movement tasks that were not part of the training and whether those potential improvements would be larger than improvements induced by RT. We performed two training interventions with two groups of healthy older adults: a traditional 12-week RT program and a 3-week PBT program consisting of support-surface perturbations of standing balance. Reactive balance performance during standing and walking as well as a set of neuro-muscular properties to quantify muscle strength, sensory and motor acuity, were assessed pre- and post-intervention. We found that both PBT and RT induced training specific improvements, i.e., standing PBT improved reactive balance during perturbed standing and RT increased strength, but neither intervention affected reactive balance performance during perturbed treadmill walking. Analysis of the reliance on different balance-correcting strategies indicated that specific improvements in the PBT group during reactive standing balance were due to adaptations in the stepping threshold. Our findings indicate that the strong specificity of PBT can present a challenge to transfer improvements to fall prevention and should be considered in the design of an intervention. Next, we found that lack of improvement in muscle strength did not limit improving reactive balance in healthy older adults. For improving our understanding of generalizability of specific PBT in future research, we suggest performing an analysis of the reliance on the different balance-correcting strategies during both the training and assessment tasks.

## Introduction

About one third of adults over the age of 65 fall each year (Tinetti et al., 1988). Approximately 30% of falls result in injuries requiring medical attention, with fractures occurring in ~10% of these falls (Berry and Miller, 2008). Therefore, targeted interventions based on a good understanding of the circumstances in which falls occur and the underlying neuro-muscular mechanisms of increased fall risk are of great importance. Both resistance training (RT) (Liu-Ambrose et al., 2004) and perturbation-based training (PBT) (Mccrum et al., 2017) have been proposed and applied as interventions to reduce fall incidence in older adults. RT aims to improve or restore muscle strength by performing repetitive contractions against an external resistance. In previous studies, RT interventions have at most resulted in limited improvements in balance performance (Orr et al., 2008) and fall incidence (Faber et al., 2006; Fairhall et al., 2014). PBT is a more recent training paradigm and aims at improving reactive balance control by applying repeated unpredictable mechanical perturbations, typically during a small number of training sessions (Dijkstra et al., 2015; Mccrum et al., 2017). PBT is considered a promising intervention as it has been shown to improve balance during the trained task (Pai and Bhatt, 2007; Dijkstra et al., 2015) and seems to reduce fall risk (Pai and Bhatt, 2007; Mansfield et al., 2015; Gerards et al., 2017; Mccrum et al., 2017). From the principle of training specificity, improvements in reactive balance control in the task trained during PBT are expected. However, the adaptations in balance-correcting mechanisms that underlie improvements in reactive balance control after PBT and RT are not well-understood. Moreover, it is unclear whether PBT and RT induce adaptations in balance control that generalize to movement tasks that were not part of the training.

The most common circumstances in which falls occur in older adults are incorrect body-weight shifts, slips and trips (Berg et al., 1997; Robinovitch et al., 2013), indicating that maintaining reactive balance performance with age is key to prevent falls. Reactive balance is the skill to perform a balance-correcting response following a perturbation in order to avoid a loss of balance (Woollacott and Shumway-Cook, 2005). From a mechanical point of view, three strategies constitute a reactive balance-correcting response each requiring different muscular coordination (Hof, 2007; Halvorsen, 2010) (**Figure 2**): (1) a center-of-pressure (COP) or ankle strategy relies on a change in the ankle torque, shifting the COP location under the foot, in order to return the center-of-mass to the equilibrium position while the body sways around the ankle joint as a simple inverted pendulum; (2) a hip or inertial strategy relies on counter-rotation of body segments with respect to the center-of-mass (COM) to dissipate the change in angular momentum induced by the perturbation; and (3) a step strategy increases the base-of-support by taking a step. The timely and appropriate combination of these balance-correcting strategies determines reactive balance performance.

Older adults use these balance-correcting strategies differently than younger adults in response to perturbations during standing and walking. As compared to young adults, older adults rely less on COP (Gruben and Boehm, 2014) and inertial strategies (Afschrift et al., 2017) to attenuate the effect of perturbations during both standing (Runge et al., 1999; Jensen et al., 2001) and walking (Afschrift et al., 2019). Hence, older adults initiate stepping strategies at lower balance disturbances (Pai et al., 1998) resulting in more frequent use of stepping strategies and adapt step length more in response to perturbations during walking (Afschrift et al., 2019) than young adults. The lower stepping threshold during standing (Pai et al., 1998) and increased reliance on stepping strategies during walking (Afschrift et al., 2019) might suggest an age-related change in a common mechanism underlying both standing and walking balance. If that were the case, a training intervention that induces adaptation in that common mechanism would thus reduce the use of stepping strategies during both standing and walking.

Age-related changes in balance control might have multiple origins as the timely and appropriate implementation of balance-correcting strategies depends on the accuracy of integrated sensory information, the transformation of this information into motor commands and finally the functional capacity of the motor system that executes these commands (Pasma et al., 2014). Several age-related changes in sensorimotor function have been associated with decreased reactive balance performance and/or increased fall risk: reduced muscle function (e.g., maximal strength, rate of force development) (Morley, 2008), decline of visual (Lord and Dayhew, 2001), vestibular (Herdman et al., 2000), and proprioceptive acuity, increased neuromuscular noise (Singh et al., 2012), and decreased peripheral nerve conduction velocity (Pasma et al., 2014). In addition, several studies suggest that altered sensorimotor transformations, i.e., the transformation of sensory information into motor commands, can explain age-related changes in postural control (Bugnariu and Fung, 2006; Yeh et al., 2014) and other tasks such as reaching (Goodman et al., 2020). Hence, we expect that interventions might improve reactive balance control by (a) increasing acuity of sensory information, (b) inducing adaptations in sensorimotor transformations (Safavynia and Ting, 2013; Welch and Ting, 2014; Afschrift et al., 2021), or (c) improving capacities of the motor system (e.g., increasing muscle strength).

RT interventions have been successful in increasing muscle strength but have induced limited improvements in reactive balance performance (Hess et al., 2006) and reductions of fall risk (Faber et al., 2006; Cadore et al., 2014; Fairhall et al., 2014; De Labra et al., 2015). Age-related decreases in muscle strength and rate of force development potentially limit the COP strategy (Robinovitch et al., 2002; Hess et al., 2006) and impair the potential to quickly increase the base of support (BOS) when taking a step (Karamanidis et al., 2008). Higher maximal strength has been associated with better reactive balance performance during non-stepping responses in response to perturbations of standing (Mackey and Robinovitch, 2006) and lower fall risk (Melzer et al., 2004; Lin and Woollacott, 2005; Pijnappels et al., 2008; LaRoche et al., 2010; Cattagni et al., 2014; Gadelha et al., 2018). Some studies found similar associations between the rate of force development and balance performance (Pijnappels et al., 2008), whereas others did not (LaRoche et al., 2010; Kamo et al., 2019). Yet RT has not consistently led to improved reactive balance and it is unclear whether RT induced improvements in muscle strength lead to more efficient application of the COP strategy in healthy older adults.

PBT is a promising approach to improve reactive balance, but the mechanisms underlying improvements in reactive balance are not yet understood. PBT has induced improvements in reactive balance for the task being trained within a session (Sakai et al., 2008; Bierbaum et al., 2010; Tanvi et al., 2012) or after a couple of sessions (Dijkstra et al., 2015; Alizadehsaravi et al., 2021). Improvements in the trained task have been shown to be retained (Pai and Bhatt, 2007), and there is even some evidence of decreased fall risk following PBT (Mansfield et al., 2015; Gerards et al., 2017; Mccrum et al., 2017). Although PBT interventions yield some general exercise, they are not expected to introduce peripheral adaptations in skeletal muscle that lead to higher muscle strength. Such changes in muscle strength are especially unlikely because PBT is typically limited to a couple of days or weeks (Mansfield et al., 2007; Mccrum et al., 2017). Similarly, physiological changes at the cell level improving the acuity of sensory and motor systems are unlikely at this time scale (Aman et al., 2015). More likely, PBT affects the sensorimotor transformations that govern how the different balance-correcting strategies are combined. However, to the best of our knowledge, no studies describe how PBT alters the application of balance-correcting strategies.

In addition, it is unclear whether alterations in balance control during the trained task generalize to other tasks. A limited number of studies indicate improvements in reactive balance during tasks that were not trained. Parijat and Lockhart (2012) showed that practicing a slip-perturbation during walking, applied by means of translating a movable part of the walkway upon heel strike, improved balance when walking on a slippery surface. In this study however, the training exercises were very similar to the actual task performed during the pre- and post-training assessment. Kurz et al. (2016) demonstrated that a training intervention based on unexpected perturbations during walking improves the ability to voluntarily step rapidly in older adults. This is a generalization of reactive balance to a different task, but the non-trained task is not challenging reactive balance directly. Next, Gimmon et al. (2018) showed that a training intervention based on unexpected perturbations during walking induced adaptations in the nominal gait kinematics, but also here it is unclear whether reactive balance performance improved. Studies by Arampatzis et al. (2011) and Bierbaum et al. (2013) showed that older adults who trained on the hip and stepping strategy mechanisms in a functional way improved in an untrained lean-and-release task and untrained perturbed walking task, respectively. The lean-and-release task was again similar to the training exercises and so limited insight on the generalizability of the improved mechanisms to other locomotion tasks is provided. In both studies it is unclear which adapations in the application of the balance-correcting strategies occurred and how these were implemented in order to improve performance in the untrained task.

In this study, we evaluated whether PBT using support-surface perturbations during standing as the training task improved reactive balance performance during perturbed walking and walking on a narrow beam more than RT in healthy older adults. In addition, we explored the effect of both training paradigms on the application of balance-correcting strategies and on sensorimotor acuity. We performed two training interventions with two groups of healthy older adults: a 12-week RT and a 3-week PBT consisting of support-surface perturbations of standing balance. The dosages of both training interventions were in line with common dosages for RT and PBT (Latham et al., 2004; Pai and Bhatt, 2007; Mccrum et al., 2017). For both interventions, the chosen dosage has been shown to induce specific improvements in previous studies (Latham et al., 2004; Dijkstra et al., 2015; Mccrum et al., 2017). Reactive balance performance during standing and walking as well as a set of neuro-muscular properties were assessed pre- and post-intervention. We hypothesized that the training programs would induce specific improvements, where PBT during standing would improve reactive balance performance during standing and RT would improve muscle strength. Therefore, we tested whether:

(1) step incidence in response to perturbations of standing balance at specific perturbation magnitudes decreased more after PBT than after RT;
(2) maximal isometric strength improved after RT, but not after PBT.

We hypothesized that standing PBT outperformed RT in improving reactive balance control during tasks that were not included in the training. More specifically we hypothesized that:

(3) step length corrections during perturbed walking decreased more after PBT than after RT;
(4) the distance covered in a narrow-beam walking task increased more after PBT than after RT.

We performed an explorative analysis to describe adaptations in the use of the different balance-correcting strategies to perturbed standing. In addition, we explored whether RT and PBT induced changes in sensory and motor acuity.

## Methods

**Figure 1:**
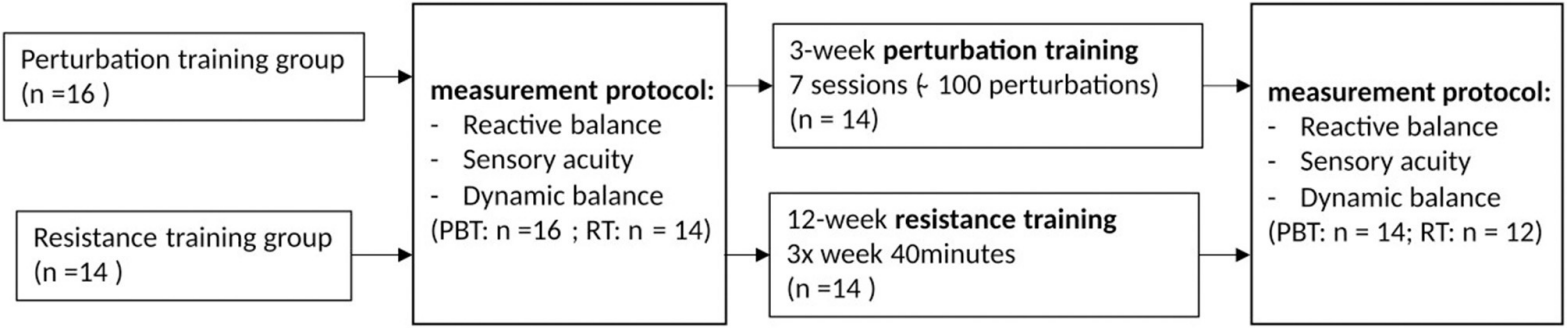
Flow chart of intervention study with pre- and post-intervention measurements.

Two groups of healthy older adults (>65 y) participated in a longitudinal study that consisted of a training intervention with pre- and post-intervention assessment sessions (Figure 1). Participants had not been enrolled in similar training programs previously. Assessors were for practical reasons not blinded, which is a limitation of the presented study. The perturbation-based training (PBT) group consisted of 16 individuals that followed a perturbation-based balance training program during 3 weeks. The resistance training (RT) group consisted of 14 individuals that were part of a larger group participating in a study that was focused on observing effects of omega-3 supplementation during a 12-week RT training program on muscle strength, and muscle anabolic insensitivity (Dalle et al., 2021). Those that indicated their interest, combined the RT training program with the assessment sessions of the present study. The PBT group was not omega-3 supplemented, but it was assumed that this difference could not directly account for any of the differences in reactive balance performance between groups. Sample sizes were based on previous studies that demonstrated specific improvements for both resistance training (Latham et al., 2004) and perturbation-based training (Dijkstra et al., 2015). A limitation was that no sample size estimation based on expected effect sizes for non-specific improvements was performed as we did not dispose of estimated effect sizes for these potential improvements.

To qualify the group of older adults as healthy, they performed a 5x sit-to-stand test (Cesari et al., 2009) and a test measuring handgrip strength (Dodds et al., 2014) and filled out the Fall Efficacy Scale-International (FES-I) questionnaire (Yardley et al., 2005; Kurz et al., 2016). None of the included older adults scored under the cut-off points for both the 5x sit-to-stand test or hand grip strength test that would indicate frailty (Cruz-Jentoft et al., 2019). None of the participants had a “high concern” score for the FES-I. Participants that suffered from musculoskeletal injury or pathologies that could impair balance were excluded.

From the PBT group, two participants did not complete the full intervention (one participant due to COVID-19 impact and another participant because they were diagnosed with health issues affecting balance control during the intervention and therefore no longer met the inclusion criteria). From the RT group, one participant did not participate in the post-intervention assessment and one participant was excluded due to lower back pain problems during the post-intervention assessment.

The assessment performed pre- and post-intervention served to quantify reactive balance performance and balance-correcting strategies during standing and walking, muscle strength and sensory acuity. We quantified standing reactive balance performance and strategies (CAREN platform, Motek); walking reactive balance performance and strategies (instrumented treadmill); maximal isometric knee-extension torque (Biodex); motor acuity (Biodex); sensory acuity (NeuroCom Balance Master); and dynamic balance [beam walking test (Mansfield et al., 2015)].

### Reactive Balance

#### Standing Reactive Balance Protocol

**Figure 2:**
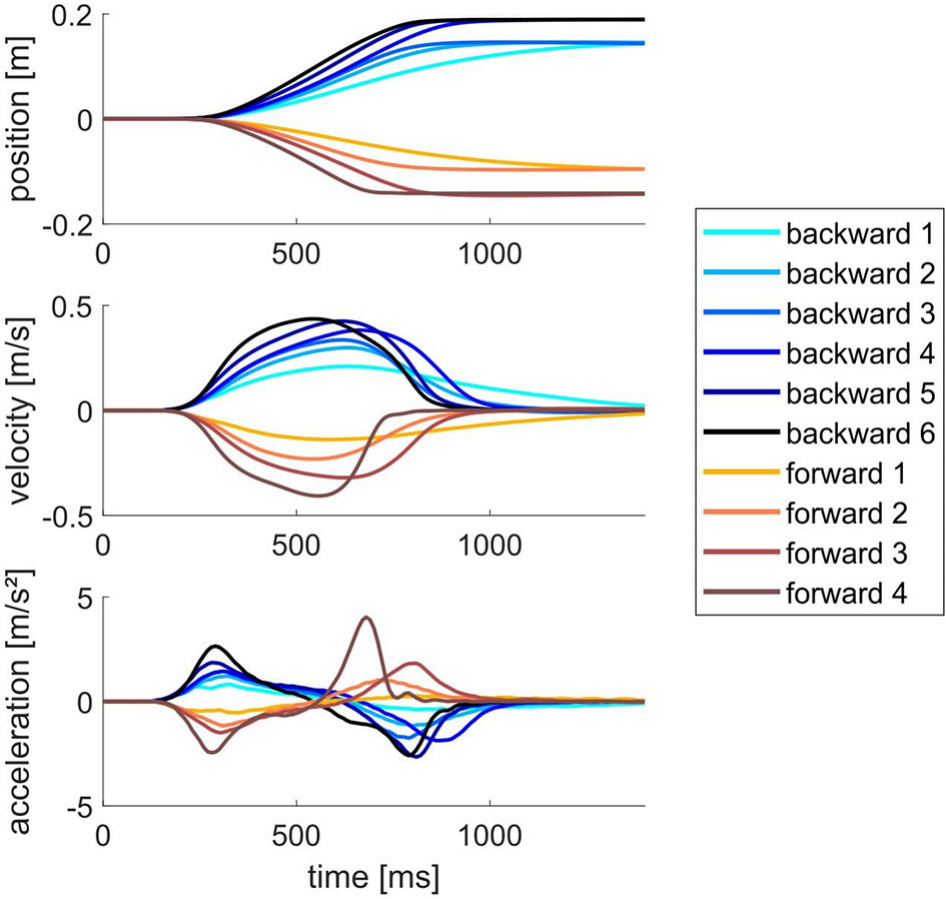
Position, velocity and acceleration profiles of the platform for the six backward and four forward support-surface translations.

To assess reactive balance performance during standing we quantified the step incidence for specific perturbation directions and magnitudes, where perturbations were applied as support-surface translations using a CAREN platform (Motek Medical, Netherlands) (Van Wouwe et al., 2020). Participants stood barefoot on the movable platform with their feet at shoulder width looking forward and wore a safety harness to catch them in case of a loss of balance. Participants were instructed to maintain balance without taking a step when perturbed and were allowed to move their arms freely. If the perturbation elicited a stepping response, participants were instructed to return themselves to their original position before the next perturbation. To standardize foot placement, the heel position was marked on the platform. Participants received support-surface perturbations in six directions: anterior and posterior translations, lateral left and right translations, and pitch rotations in two directions inducing either ankle plantar- or dorsiflexion. The protocol consisted of a familiarization part and a randomized part. Subjects were first familiarized with the motion of the platform while being informed on the direction of the upcoming perturbation. During this familiarization, perturbations were applied with progressively larger magnitudes until subjects needed to take a step, which ended familiarization with the specific perturbation direction. The first perturbation magnitude that induced a step response was the highest magnitude included in the second, randomized part of the protocol. When no step response was evoked at the highest magnitude, all perturbations for that direction were included. Up to six different perturbation magnitudes were presented for posterior translations, whereas up to four different perturbation magnitudes were presented in the other directions (Figure 2). Next, during the randomized part of the protocol, each perturbation condition was applied five times in random order. Perturbations were provided in random order to minimize anticipatory postural adjustments. We quantified step incidence for the backward and forward platform translations at each presented perturbation intensity.

#### Walking Reactive Balance Protocol

To assess reactive balance performance during treadmill walking we quantified changes in step length in response to treadmill belt accelerations and decelerations with four different magnitudes (Afschrift et al., 2019). The protocol is the same as the part of the protocol with sagittal perturbations in the study by Afschrift et al. (2019). Participants walked with shoes on an instrumented treadmill and wore a safety harness to prevent falling in case of a loss of balance. The protocol consisted of a familiarization part and unperturbed walking part and a randomized perturbation part (perturbed walking). During the familiarization part subjects walked on the treadmill until they were comfortable at the baseline speed of 1.0 m/s, a speed at which healthy older adults are comfortable to execute the whole protocol (Afschrift et al., 2019). During the unperturbed walking part, subjects got used to treadmill walking at a speed of 1.0 m/s for 2 min and their baseline walking pattern was collected. During the randomized perturbation part, subjects were exposed to 16 different perturbations, belt accelerations or decelerations with four different magnitudes applied at two different instants in the gait cycle. Perturbations were applied immediately after left heel strike (7.5% gait cycle, first double support), and during late stance (37.5% gait cycle). These timings and perturbation magnitudes were motivated based on the work of Afschrift et al. (2019) that showed largest differences between young and older adults for these perturbations within a larger set of timings at which perturbations were applied. The order of the perturbations was randomized. A next perturbation was applied when the operator indicated that it was safe to apply a perturbation to the participant and the participant had reached steady state walking, for which the standard deviation of the stride time of the last five strides had to be below 0.05 s.

#### Motion Capture Data During Standing and Walking

Motion capture data was collected during both reactive standing and walking balance assessments. Subjects were instrumented with 33 reflective markers on anatomical landmarks (full body plug-in-gait) and cluster markers on the left and right shanks and thighs. Platform motion during perturbations of standing was measured using three markers. The marker trajectories were captured using seven and fifteen Vicon cameras during standing and walking, respectively, at a frequency of 100 Hz. Both the treadmill and CAREN platform were instrumented with two force plates, measuring contact forces and moments between the subjects and the support-surface at 1,000 Hz. A static trial in anatomical position was acquired before starting the experiments.

Data was preprocessed to get joint, COM, and COP kinematics and joint kinetics. All marker trajectories were labeled in Vicon Nexus 2.4. Generic musculoskeletal models (gait2392 - OpenSim 3.3) were scaled based on the subject mass and anatomic marker positions acquired during the static trial (Delp et al., 2007; Seth et al., 2018). Joint angles were computed using OpenSim's Inverse Kinematics tool (OpenSim3.3). Finally, OpenSim's Body Kinematics tool was used to compute segment and whole body kinematics. Center of pressure (COP) locations were derived from the forces and moments recorded by the force plates. Joint kinetics were computed using OpenSim's Inverse Dynamics tool (OpenSim3.3), with the scaled musculoskeletal models, force plate data and joint kinematics as input. A correction of the force plate data was performed to remove forces and moments registered due to the inertia of the force plate (Roberts et al., 2019). We corrected for these forces and moments by subtracting the forces and moments registered while the platform was moving without any load on it from the data acquired with the subject on the platform (Van Wouwe et al., 2020).

#### Outcome Variables for Reactive Balance Performance During Standing and Walking

During perturbed standing, step incidence within each perturbation type was computed by detecting trials in which the vertical ground reaction force (GRF) was below 10 N during more than 50 ms. The step incidence at the largest perturbation magnitude applied pre-intervention was used as outcome variable for both pre -and post-intervention assessments.

During perturbed walking, step length was computed as the sagittal plane distance between the left and right ankle joint center at heel-strike, where heel-strike was defined as the first instant at which the vertical GRF was higher than 10 N after swing phase. Rather than using the step length during unperturbed walking as reference we computed the reference step length as the average length over all last steps before a perturbation during perturbed walking. We did this because some subjects adapted their step length during perturbed walking compared to the familiarization part of the protocol.

#### Outcome Variables to Quantify Reliance on Balance-Correcting Strategies

To explore adaptations in sensorimotor transformation that lead to changes in the application of the different balance-correcting strategies, we quantified the strategies as described in the following paragraphs.

The reliance on the COP strategy was quantified by the feedback gain (*K*_*COP*_) that linearly relates the deviation of the delayed (100 ms) anteroposterior extrapolated COM position (Δ*xCOM*) with the corrective ankle joint torque (Δ*T*_*A*_) (Afschrift et al., 2021):


$$\begin{array}{l} \Delta T_{A}\left(t\right)=\;K_{COP}\Delta xCOM\left(t-100ms\right)\;with\;t=50\ldots 200ms \end{array}$$

We reduced the feedback model used in (Afschrift et al., 2021), to the *xCOM* rather than both COM position and velocity in order to only have a single feedback gain quantifying the sensorimotor transformation. During standing, deviations of *T*_*A*_ and *xCOM* were computed with respect to *T*_*A*_and *xCOM* at perturbation onset. All quantities and results were non-dimensionalized using COM height during quiet standing (*l*_max_), the gravitational acceleration (*g*) and body mass (*m*). COM positions were normalized by *l*_max_ and torques by *mgl*_max_ (Gruben and Boehm, 2014). Subject-specific feedback gains were estimated from the measured kinematics and joint moments by solving a robust least squares regression (MATLAB R2020a; “*lmfit”* with robust fitting option), pooling the data of all anterior-posterior perturbation trials (Afschrift et al., 2021). An increase in *K*_*COP*_ indicated that the subjects increased their reliance on COP strategies.

The reliance on a hip strategy was quantified by the relation between the *xCOM* position 300 ms after perturbation onset (*xCOM*_300*ms*_) and the maximal trunk lean angle θ_*trunk*, max_ during non-stepping responses *K*_*hip*_ (Van Wouwe et al., 2020). The maximal trunk lean angle θ_*trunk*, max_ is a measure of the reliance on a hip strategy for a specific trial. However, θ_*trunk*, max_ depends on the perturbation magnitude and, as we recently demonstrated, the initial posture of the subject (Van Wouwe et al., 2020). *xCOM* 300 ms after perturbation onset (*xCOM*_300*ms*_) captures the effect of both the perturbation magnitude and initial posture (Van Wouwe et al., 2020). Therefore, the relation between θ_*trunk*, max_ and *xCOM*_300*ms*_ established based on several perturbation trials better quantifies an individual's reliance on a hip strategy than θ_*trunk*, max_. To allow for better comparison between subjects *xCOM*_300*ms*_ was normalized by the subject-specific BOS (*xCOM*_300*ms*_/*BOS*). The BOS at perturbation onset was computed as the horizontal distance from the toes to the ankle joint, for the anterior direction, and from the heel to the ankle joint for the posterior direction. Subject specific robust linear regression models were generated for each individual with θ_*trunk*, max_ as outcome variable and *xCOM*_300*ms*_/*BOS* as predictor variable. The model had a fixed intercept and the slope coefficients were variable with respect to the categorical variable time (pre vs. post). An increase in slope coefficient (*K*_*hip*_) indicated that the subjects increased their reliance on hip strategies.

The reliance on a stepping strategy was quantified by the stepping threshold, which was defined as the maximal extrapolated COM excursion in non-stepping trials. We normalized again by the base-of-support *xCOM*_max, *non*−*stepping*_/*BOS*. This outcome variable captures how strongly a subject's balance was disturbed before they initiated a stepping response. For each non-stepping trial, we computed the largest within trial *xCOM* values. For each subject, pre -and post-intervention, we computed the mean over the three largest values *xCOM*_max, *non*−*stepping*_/*BOS*. An increase of *xCOM*_max, *non*−*stepping*_/*BOS* post-intervention indicated that subjects increased their stepping threshold and thus relied less on a stepping strategy.

### Muscle Strength

Muscle strength was assessed for the knee-extensor muscles on a Biodex System 4 PRO dynamometer (Shirley, NY). Subjects were seated on the Biodex equipment with their knee at a 90° flexion angle with the back support in fully upright position. Subjects performed three maximal voluntary isometric knee extension contractions of 3 s with 1-min rest between trials. The torque was measured at 1,000 Hz. From these three trials the maximal voluntary isometric knee extension torque [MVIKT (Nm)], normalized by the body weight, was computed.

### Motor Acuity

Motor acuity was tested by measuring force fluctuations during submaximal isometric knee extension (Singh et al., 2010, 2012, 2013). Force fluctuations were tested at 15 and 20% of the measured maximal isometric knee extension torque. To assess force fluctuations three different torque tracking tasks where executed three times in random order. Task 1 and 2 consisted of generating a constant torque for 15 s at, respectively, the 15 and 20% level, task 3 consisted of tracking a ramp-up torque from the 15 to 20% level during 15 s. The target torque profile was displayed on a monitor and participants were instructed to match the torque level as well as they could for the duration of each test by generating knee-extension torque at 90° flexion angle. The torque generated by the subjects (*GT*) was overlaid in real-time on the target torque (*TT*).

The recorded torque profiles were first low-pass filtered using a fourth-order low-pass Butterworth filter with a cut-off frequency of 25 Hz (Singh et al., 2013). Force fluctuation for each of the trials was computed using the normalized standard deviation (SD) of the absolute error (NSAE) between the target and generated torque during the middle 10 s of each trial (Christou and Carlton, 2001; Singh et al., 2010):


$$\begin{array}{l} NSAE=\;SD\left(TT\left(t\right)-GT\left(t\right)\right)/mean\left(GT\left(t\right)\right) \end{array}$$

A composite score to quantify motor acuity for each subject was then computed by taking the average over the nine trials.

### Sensory Acuity

Sensory acuity in the context of balance control was tested by performing a sensory organization test (SOT) (Nashner and Peters, 1990) on a NeuroCom Balance Master, yielding a composite score, subscores to quantify visual, vestibular and proprioceptive acuity and a preference score that quantifies the subjects' ability to organize and select the appropriate sensory information to maintain balance (Ford-Smith et al., 1995). The composite score was the main outcome measure, but subscores were analyzed as well.

### Dynamic Balance

To quantify non-reactive dynamic balance control, subjects performed a narrow beam walking task (Speers et al., 1998; Sawers and Hafner, 2018a). Subjects were asked to walk on a narrow 3.66 m long beam (width: 2 cm; height: 2 cm) six times (Sawers and Hafner, 2018a). The only instructions were to start with one foot on the beam with the heel lining up with the start of the beam and to maximize the distance covered on the beam without touching the ground. The distance was measured between the start of the beam and the position where one of the feet touched the ground or the end of the beam if subjects touched the ground beyond the end of the beam. An overall score was computed by summing the distances of all six trials and dividing this sum by the length of the beam (21.96 m), yielding a percentage score of the maximum distance that could have been covered. Before their first measured trial, subjects performed four familiarization trials. Subjects were free to use their arms.

### 12-Week Resistance Training

The 12-week supervised RT program for the lower limbs consisted of three 40-min sessions per week (Dalle et al., 2021). Each training session consisted of a 10-min warm-up on a bicycle ergometer at low intensity followed by the RT program. The RT program consisted of a leg press exercise, a leg extension exercise and calf raises. During the first 6 weeks, participants performed two sets of 12–15 repetitions at about 70% of the one repetition maximum with 1 min rest between sets and 2 min rest between exercises. During the last 6 weeks, participants completed three sets of 10–12 repetitions at ~80% of the one repetition maximum. Participants performed these exercises at moderate velocity, i.e., 3 s for the concentric and eccentric phase.

### 3-Week Standing Perturbation Training

The 3-week supervised PBT program consisted of 7 sessions in total excluding the assessment sessions. Participants stood on a movable platform and received unpredictable support-surface translations. The participants were instructed to maintain balance without taking a step. About 100 perturbations were performed each session. Similar to during assessment, support-surface perturbation were applied randomly in six directions. For each perturbation direction, different magnitudes were applied depending on the participant's level. The maximal perturbation magnitude for each perturbation direction during the first training session was determined during the familiarization part of the pre-intervention assessment. The stepping frequency for each perturbation type and magnitude was determined during each session. Based on this information, the perturbation magnitudes and number of repetitions for each perturbation magnitude for the next session were determined to increase the difficulty each session. When subjects exhibited step incidence below 25% for the largest included perturbation magnitude in a specific direction, the next session contained a perturbation with larger magnitude for the same direction. The number of perturbations for each magnitude within a perturbation direction was such that higher magnitudes were applied more than lower magnitudes.

### Statistical Analysis

An overview of all outcome variables is provided in Table 1. For the statistical analysis we reported data as means ± SD. Normality of data was tested by applying a Shapiro-Wilkes test for all outcome variables for within intervention group pre- to post-intervention differences in outcome variables. Repeated-measures ANOVA (Matlab R2020a “ranova”) with time (pre- vs. post-intervention) as within-subject factor and intervention group (RT vs. PBT) as between-subject factor was performed to test for time (pre- vs. post-intervention) and time^*^intervention interaction effects. *p*-value corrections for violations of compound symmetry within the repeated-measures ANOVA model were performed. When a significant interaction effect was observed, paired *t*-tests within intervention groups were executed to detect changes pre- to post-intervention within intervention groups.

**Table 1:** Description of outcome variables.

**Outcome variable**	**Measured for/by**	**Quantifies**
*Step incidence*	Anterior and posterior perturbations of standing with different perturbation magnitudes	Reactive standing balance performance
*Step length correction*	Anterior and posterior perturbations of walking with different magnitudes at different instances of the stance phase	Reactive walking balance performance
*K_COP_*	Anterior and posterior perturbations of standing with different perturbation magnitudes	Reliance on COP strategy: sensorimotor transformation from COM kinematics to ankle torque during standing
*K_hip_*	Anterior and posterior perturbations of standing with different perturbation magnitudes	Reliance on hip strategy: relation between *xCOM_300ms_ and θ_trunk, max_*
*xCOM_max, non−stepping_/BOS*	Anterior and posterior perturbations of standing with different perturbation magnitudes	Reliance on stepping strategy: maximum *xCOM* excursion withstanded without initiating a step response
*Maximal voluntary isometric knee-extension torque (MVIKT)*	Maximum over three trials	Knee-extensor strength
NSAE of force fluctuations	Averaged over nine torque tracking tasks on the Biodex dynamometer	Motor acuity
*SOT composite and subscores*	Sensory organization test	Sensory acuity and organization during balance control
*Beam walking score*	Averaged over six trials of narrow-beam walking	Dynamic balance

## Results

At baseline the resistance training group had a MVIKT that was 20% lower than the perturbation training group (Table 2). For all other outcome parameters and for age, body mass, length and BMI the two groups did not differ. Results for all outcome variables are reported in Tables 3, 4.

**Table 2:** Baseline comparison for the included groups of healthy older adults.

	**Resistance training**	**Perturbation-based training**	***p*** **-value**
Age (y)	72.2 ± 3.56	70.8 ± 4.6	0.41
Gender	7 male; 5 female	7 male; 7 female	
Body mass (kg)	73.1 ± 8.1	70.7 ± 14.0	0.60
Length (cm)	167 ± 7.3	169 ± 9.4	0.61
BMI (kg/m^2^)	26.2 ± 2.4	24.6 ± 3.0	0.15
*Step incidence (backward)*	0.79 ± 0.35	0.84 ± 0.36	0.66
*Step incidence (forward)*	0.87 ± 0.28	0.93 ± 0.19	0.40
MVIKT *(Nm/kg)*	1.85 ± 0.38	2.32 ± 0.54	0.03^*^
NSAE (%)	1.79 ± 0.46	1.72 ± 0.39	0.10
*SOT*	68.2 ± 9.63	71.4 ± 6.79	0.32
*Beam walking (*%)	30 ± 15	36 ± 13	0.32
Δ*l_step_ (m)early stance(backward)*	0.062 ± 0.032	0.065 ± 0.017	0.70
Δ*l_step_ (m)early stance(forward)*	−0.12 ± 0.040	−0.13 ± 0.049	0.77
Δ*l_step_ (m)late stance(backward)*	0.078 ± 0.030	0.068 ± 0.029	0.48
Δ*l_step_ (m)late stance(forward)*	−0.003 ± 0.057	−0.032 ± 0.045	0.22
*K_COP_*	0.66 ± 0.20	0.65 ± 0.10	0.39
*K_hip_*	32.6 ± 35.5	35.2 ± 21.5	0.34
*xCOM_max, non−stepping_/BOS*	1.13 ± 0.19	1.14 ± 0.18	0.70

* *significance at 0.05 alpha level*.

**Table 3:** Pre -to post-training comparisons for different outcome variables that were defined a-priori.

	**Resistance training**		**Perturbation-based training**		***P*** **-value**
	**Pre**	**Post**	**Pre**	**Post**	
*Step incidence (backward)*	0.79 ± 0.35	0.63 ± 0.41^*^ −0.25 ± 0.38^+^	0.84 ± 0.36	0.34 ± 0.40^**^ −0.50 ± 0.46	*p* < 0.001^a^ *p* = 0.0175^b^
*Step incidence* *(forward)*	0.87 ± 0.28	0.71 ± 0.37−0.15 ± 0.33	0.93 ± 0.19	0.58 ± 0.35^**^ −0.35 ± 0.33	*p* < 0.001^a^ NS (*p* = 0.13)^b^
MVIKT *(Nm/kg)*	1.85 ± 0.38	2.15 ± 0.38^***^ 0.30 ± 0.17^+++^	2.32 ± 0.54	2.27 ± 0.43−0.043 ± 0.24	*p* = 0.0044^a^ *p* < 0.001^b^
NSAE (%)	1.79 ± 0.46	1.66 ± 0.33−0.14 ± 0.33	1.72 ± 0.39	1.51 ± 0.40−0.21 ± 0.38	*p* = 0.0198 ^a^ NS (*p* = 0.61)^b^
*SOT*	68.2 ± 9.63	73.2 ± 6.58^*^ 5.00 ± 6.61	71.4 ± 6.79	76.1 ± 6.73^**^ 4.64 ± 5.00	*p* < 0.001^a^ NS (*p* = 0.87)^b^
*Beam walking* (%)	30 ± 15	33 ± 202.4 ± 11	36 ± 13	42 ± 17^**^ 6.0 ± 6.4	*p* = 0.021 ^a^ NS (*p* = 0.31)^b^
Δ*l_step_ (m)early stance* *(backward)*	0.062 ± 0.032	0.056 ± 0.039	0.065 ± 0.017	0.027 ± 0.037	NS (*p* = 0.30^a^) NS (*p* = 0.86^b^)
Δ*l_step_ (m)early stance* *(forward)*	−0.12 ± 0.040	−0.12 ± 0.024	−0.13 ± 0.049	−0.13 ± 0.035	NS (*p* = 0.96^a^) NS (*p* = 0.97^b^)
Δ*l_step_ (m)late stance* *(backward)*	0.078 ± 0.030	0.061 ± 0.024	0.068 ± 0.029	0.070 ± 0.023	NS (*p* = 0.30^a^) NS (*p* = 0.16^b^)
Δ*l_step_ (m)late stance* *(forward)*	−0.003 ± 0.057	−0.015 ± 0.06	−0.032 ± 0.045	−0.005 ± 0.046	NS (*p* = 0.59^a^) NS (*p* = 0.18^b^)

*All statistical model residuals were normally distributed, and so repeated-measures ANOVA and parametric tests were performed for the different outcome variables. Values are means ± SD. Where relevant the changes from pre- to post-assessment values are reported on the second line in the post-assessment column. p-values obtained by repeated-measures ANOVA with time as within-subject effect and training as between-subject effect.*

a *Main time-effect (pre -to post-training).*

b Time*intervention interaction effect.

*** *p < 0.001,*

** *p < 0.005,*

* *p < 0.05 significant difference within group from pre -to post-training. ^+++^p < 0.001, ^+^p < 0.05 significant difference in pre- to post-training changes between PBT and RT groups*.

**Table 4:** Exploratory outcome variables.

***K*** ***_COP_***	**0.66 ± 0.20**	**0.63 ± 0.14−0.036 ± 0.14**	**0.65 ± 0.10**	**0.63 ± 0.17−0.022 ± 0.15**	**NS (*p =* 0.59)^a^** **NS (*p =* 0.81)^b^**
***K*** ***_hip_***	**32.6 ± 35.5**	**32.8 ± 30.8**	**35.2 ± 21.5**	**43.2 ± 29.6**	**NS (*p* = 0.0813^a^)** **NS (*p* = 0.096^b^)**
***xCOM*** ***_max, non−stepping_/BOS***	**1.13 ± 0.19**	**1.18 ± 0.136.2 ± 20.1%^+^**	**1.14 ± 0.18**	1.27 ± 0.17^**^ **13.6 ± 16.3%**	***p*** ***= 0.0015^a^*** *p* = 0.043^b^

a *Main time-effect (pre -to post-training).*

b Time*intervention interaction effect.

** *p < 0.005 significant difference within group from pre -to post-training. ^+^p < 0.05 significant difference in pre- to post-training changes between PBT and RT groups*.

### Summary of the Main Observations

**Figure 3:**
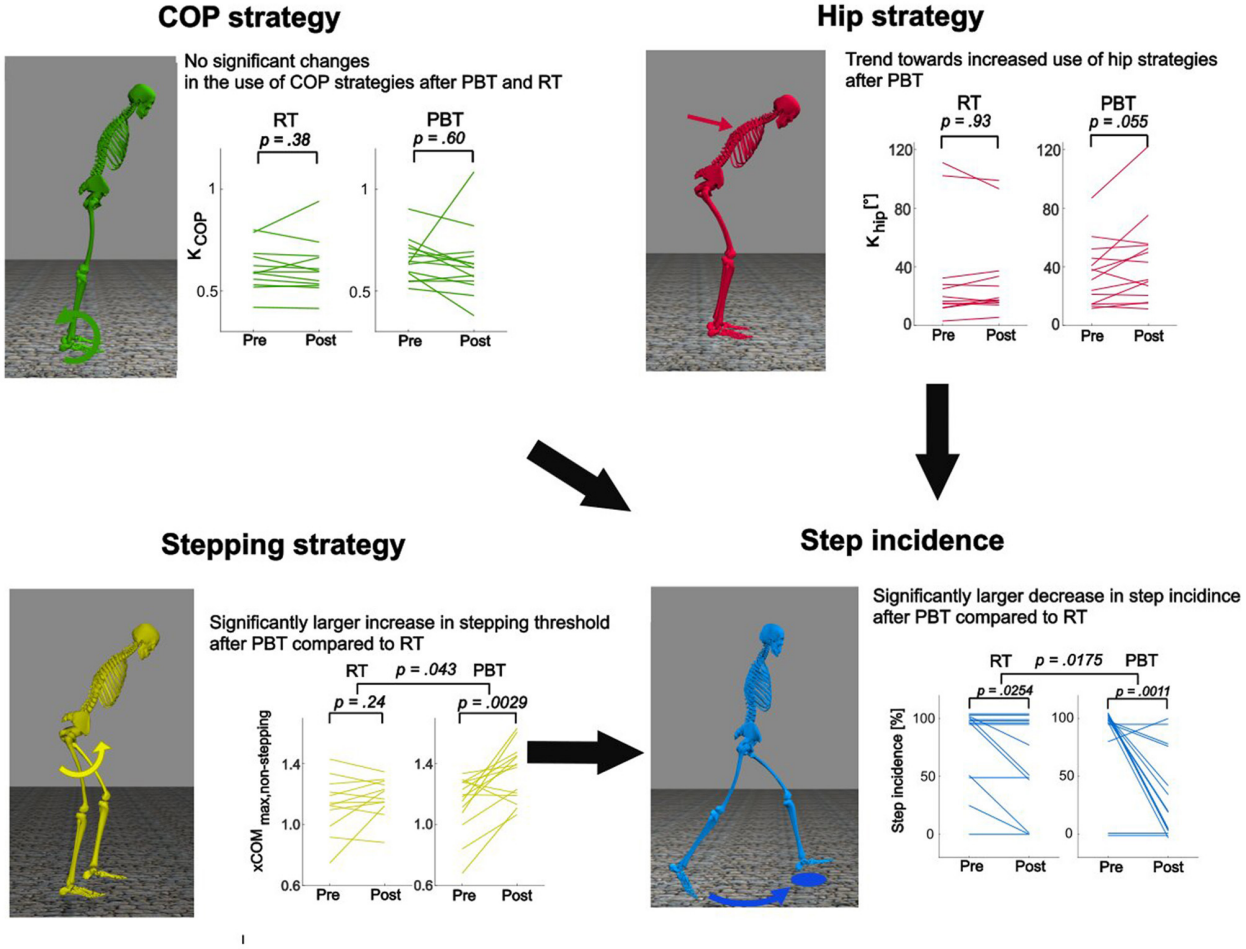
Effects of resistance training (RT) and perturbation-based training (PBT) on reactive standing balance performance and strategies. Step incidence during backward perturbations of standing decreased more after PBT than after RT. Improvements in step incidence were not explained by changes in the COP strategy defined by the sensorimotor transformation from xCOM to *T*_*A*_. A trend toward increased use of hip strategies might have contributed to the decreased step incidence. Increased reliance on hip strategies was observed in some but not all individuals. Most likely, decreased step incidence might be due to changes in reliance on step strategies quantified by a significant change in stepping threshold after PBT that was not observed after RT.

Both PBT and RT induced training specific improvements. PBT reduced step incidence during backward perturbations of standing more than RT (hypothesis 1; Figure 3), while RT increased maximal strength of the knee extensors whereas PBT did not (hypothesis 2).

Reduced step incidence after PBT could not be explained by changes in the sensorimotor transformation from xCOM to *T*_*A*_ that determine the reliance on the COP strategy (Figure 3). Exploring other potential mechanisms reveals that an increased stepping threshold might have reduced step incidence after PBT, whereas an increased reliance on hip strategies might have contributed to the reduced step incidence in part of the subjects (Figure 3).

Improvements in balance during standing after PBT do not seem to generalize to perturbed walking or narrow beam walking. Neither PBT nor RT induced changes in step length corrections during perturbations of walking (hypothesis 3). Finally, beam walking performance improved significantly after the interventions but the improvements after PBT and RT were not different (hypothesis 4).

### Reactive Balance

#### Standing Reactive Balance

Step incidence in response to backward platform translations (backward perturbations) of standing presented both a time main effect [*F*_(1, 24)_ = 22.8, *p* < 0.001] and time^*^intervention interaction effect [*F*_(1, 24)_ = 6.5, *p* = 0.0175]. Both RT [*t*_(11)_ = 2.55, *p* = 0.0254] and PBT [*t*_(13)_ = 4.20, *p* = 0.0011] induced a significant reduction in step incidence but PBT induced a significantly larger pre- to post-intervention reduction in step incidence, confirming hypothesis 1 for backward perturbations.

Step incidence in response to forward platform translations (forward perturbations) presented a time main-effect [*F*_(1, 24)_ = 16.1, *p* < 0.001], but not a time^*^intervention interaction effect [*F*_(1, 24)_ = 2.5, *p* = 0.13]. Step incidence decreased significantly after the intervention, but the decrease was not different between PBT and RT.

*K*_*COP*_ during perturbed standing did not present a time main effect [*F*_(1, 24)_ = 0.29, *p* = 0.59] nor a time^*^intervention interaction effect [*F*_(1, 24)_ = 0.06, *p* = 0.81] indicating that neither intervention induced a change in the sensorimotor transformation between *xCOM and* *T*_*A*_. The subject-specific linear regression models fitted the data well with mean R-squared values over all subjects being 0.90, 0.88, 0.90, and 0.89 for the RT pre-intervention, RT post-intervention, PBT pre-intervention and PBT post-intervention data, respectively. The minimal R-squared value was 0.73. These findings indicate that PBT does not increase the reliance on COP strategies.

Reliance on hip strategies was quantified by the slope coefficient *K*_*hip*_ that linearly relates the *xCOM*_300*ms*_ and θ_*trunk*, max_. We did not detect significant changes in *K*_*hip*_, i.e., no time main-effect [*F*_(1, 24)_ = 3.3, *p* = 0.081] nor a time^*^intervention interaction-effect [*F*_(1, 25)_ = 3.0, *p* = 0.096].

PBT induced significantly larger increases in stepping thresholds than RT. *xCOM*_max, *non*−*stepping*_/*BOS* showed both a time main effect [*F*_(1, 24)_ = 12.8, *p* = 0.0015] and a time^*^intervention [*F*_(1, 24)_ = 4.6, *p* = 0.043]. *xCOM*_max, *non*−*stepping*_/*BOS* increased significantly in the PBT group [*t*_(13)_ = −3.65, *p* = 0.0029], but not in the RT group [*t*_(11)_ = −1.25, *p* = 0.23]. This indicates that older adults increase their stepping threshold after PBT but not after RT.

#### Walking Reactive Balance

Step length changes in response to perturbations did not change after PBT or RT. Step length corrections during perturbed walking did not present a time main effect nor time^*^intervention interaction effect during treadmill belt accelerations in early [*F*_time(1, 24)_ = 1.13, *p*_time_ = 0.31; $F_{\text{tim}{\text{e}}^{*}\text{intervention}\left(1,\;24\right)}$ = 0.03, $P_{\text{tim}{\text{e}}^{*}\text{intervention}}$ = 0.86] and late stance [*F*_time(1, 24)_ = 1.14, *p*_time_ = 0.30; $F_{\text{tim}{\text{e}}^{*}\text{intervention}\left(1,\;24\right)}$ = 1.17, $P_{\text{tim}{\text{e}}^{*}\text{intervention}}$ = 0.16] and during treadmill belt decelerations during early [*F*_time(1, 24)_ = 0.003, *p*_time_ = 0.96; $F_{\text{tim}{\text{e}}^{*}\text{intervention}\left(1,\;24\right)}$ = 0.002, $P_{\text{tim}{\text{e}}^{*}\text{intervention}}$ = 0.97] and late stance [*F*_time(1, 24)_ = 0.30, *p*_time_ = 0.59; $F_{\text{tim}{\text{e}}^{*}\text{intervention}\left(1,\;24\right)}$ = 1.97, $P_{\text{tim}{\text{e}}^{*}\text{intervention}}$ = 0.18]. We thus reject the hypothesis that the correction in step length decreased more after PBT than after RT (hypothesis 3).

### Maximal Strength

MVIKT presented both a time main effect [*F*_(1, 24)_ = 9.91, *p* = 0.0044] and time^*^intervention interaction effect [*F*_(1, 24)_ = 17.6, *p* < 0.001], indicating that RT induced larger improvements in normalized MVIKT than PBT, confirming hypothesis 2. RT induced significant increases of normalized MVIKT comparing pre- to post-measurements [*t*_(11)_ = 6.3, *p* < 0.001]. PBT did not result in significant increases of MVIKT from pre- to post-intervention [*t*_(13)_ = 0.67, *p* = 0.51].

### Narrow Beam Walking

The beam walking score presented a time main effect [*F*_(1, 24)_ = 6.0, *p* = 0.021] but not a time^*^intervention interaction effect [*F*_(1, 24)_ = 1.1, *p* = 0.31]. Subjects walked further on the beam post- than pre-interventions but these improvements were not significantly different between the two interventions. We thus reject our hypothesis that the distance covered in a narrow-beam walking task increased more after PBT than after RT (hypothesis 4).

### Motor Acuity

NSAE presented a time main effect [*F*_(1, 24)_ = 6.2, *p* = 0.0198] but no time^*^intervention interaction effect [*F*_(1, 24)_ = 0.27, *p* = 0.61].

### Sensory Acuity

Composite SOT scores presented a time main effect [*F*_(1, 24)_ = 313.4, *p* < 0.001] but not a time^*^intervention interaction effect [*F*_(1, 24)_ = 0.43, *p* = 0.88]. Further analysis of the SOT sub scores revealed no significant changes for the visual and somatosensory scores. The vestibular sub score presented a time main effect [*F*_(1, 24)_ = 5.4, *p* = 0.0285], but not a time^*^intervention interaction effect [*F*_(1, 24)_ = 3.4, *p* = 0.076]. The improvements in the sensory acuity task might thus be the result of a learning effect. The observed changes are not unexpected based on literature reporting on learning effects of this test (Wrisley et al., 2007; DiFrancisco-Donoghue et al., 2015).

## Discussion

PBT of standing balance did not improve balance control during non-trained walking tasks in healthy older adults and neither did RT (hypotheses 3 and 4 not confirmed). Both PBT and RT induced training specific improvements, i.e., standing perturbation training improved reactive balance during perturbed standing (hypothesis 1) and resistance training increased strength (hypothesis 2). Improvements in reactive standing balance after PBT, measured in terms of step incidence, were the result of an increased stepping threshold, possibly in combination with increased reliance on hip strategies, but not of an increased reliance on the COP strategy. The strong specificity of PBT should be considered in the design of an intervention as it might lead to limited effects on fall prevention, the ultimate purpose of such interventions. Increasing strength was not effective in improving reactive balance in healthy older adults. This suggests, in line with previous studies, that as long as muscle strength remains above a threshold, it is not the primary limiting factor for reactive balance.

### Training-Induced Alterations in Reactive Balance Strategies in Older Adults

Subjects increased their stepping threshold and tended to rely more on hip strategies after PBT, suggesting that the lower stepping threshold in older adults compared to young adults (Pai et al., 1998) can thus be increased through PBT. Similar to previous intervention studies that targeted perturbed standing (Dijkstra et al., 2015), and perturbed walking (Pai and Bhatt, 2007; Sakai et al., 2008), we found that PBT improved reactive balance performance for the trained task. Our observations suggest that the decreased step incidence was due to changes in control strategy, i.e., the translation of sensory information to motor commands, rather than changes in muscle strength or sensorimotor acuity. We indeed found that subjects increased their stepping threshold, expressed as the maximum *xCOM* excursion resisted without initiating a step response, by 13.6% on average after PBT. This indicates a change in the sensorimotor transformation that coordinates the muscles to initiate a step response at a specific threshold of balance disturbance.

The increase in stepping threshold after PBT, might be interpreted as relying on a higher risk strategy by allowing a larger disturbance of balance from equilibrium before initiating a step. The repeated PBT might decrease the fear that healthy older adults experience during the platform perturbations, which might allow them to better achieve the task goal of suppressing a step response. However, subjects' fear of falling questioned using a FES-I questionnaire showed low fear of falling both pre- and post-intervention in both groups.

Alternatively, step incidence can be reduced without changing the step initiation threshold by relying more on COP and hip strategies. On the one hand, we found limited changes in the application of COP strategies after RT and PBT. This might indicate that the COP strategy is limited by a factor that is not affected by the specific PBT or RT training. For example, intrinsic foot muscle capacities might be a factor limiting the COP strategy in older adults (Koyama and Yamauchi, 2017; Zhang et al., 2017). On the other hand, we found that some, but not all, subjects of the PBT group increased their reliance on hip strategies. When examining the subject-specific linear regression models we could observe whether specific subjects increased their reliance on hip strategies significantly. In the PBT group this was the case for eight out of fourteen subjects, in the RT this was the case for four out of twelve subjects. These findings indicate that PBT might induce an increased reliance on hip strategies in some subjects. Our previous simulation study showed that inter-subject differences in reliance on the hip strategy can be explained by differences in the trade-off between effort and stability in a group of young adults. Similar differences might be present in older adults (Van Wouwe et al., 2020) that might shift their strategy to maximize stability in exchange for increased effort. It remains to be investigated whether explicitly coaching subjects to rely more on a hip strategy, would have reduced step incidence further.

It is unlikely that improvements in reactive balance performance during standing were the result of changes in sensory, although we cannot exclude this based on our observations. We found improvements in sensory acuity as measured by SOT in both PBT and RT groups. It is unlikely that these improvements in SOT reflect changes in the sensory system given the short duration of the PBT, the focus of RT on strength, and the absence of differences between PBT and RT. Changes in SOT scores post- vs. pre-intervention might reflect a learning effect, in line with previous findings (Wrisley et al., 2007; DiFrancisco-Donoghue et al., 2015).

Although motor acuity measures improved after both PBT and RT, it is unlikely that these explain the changes in reactive balance performance. Our assessment of motor acuity was indirect as it involved a torque-tracking task and it is therefore possible that there was a learning effect. Considering the short duration of PBT since it is unlikely that physiological changes took place. In contrast, RT induced physiological changes in the muscle and might therefore have had an effect on motor acuity, yet changes after PBT and RT did not differ.

Increasing muscle strength should not be the main target when aiming to improve reactive balance in healthy older adults. RT decreased step incidence during a reactive standing balance task but to a smaller extent than PBT, which did not alter muscle strength. These findings are in line with previous intervention studies that found little or no improvements in fall risk (Faber et al., 2006; Cadore et al., 2014; Fairhall et al., 2014; De Labra et al., 2015) or reactive balance (Hess et al., 2006) following RT. It is likely that learning effects, rather than increases in strength after RT, explain the observed reduction in step incidence after RT. Indeed, the pre- and post-assessments of reactive standing balance can be considered a perturbation-based training session and PBT had a large effect on step incidence. Although healthy older adults use stepping strategies at lower perturbation magnitudes than young adults that are stronger (Pai et al., 1998), these alterations in reactive balance might not be due to reductions in strength. Simulation studies that have elicited causal relations between muscle strength and balance-correcting responses following perturbations of standing (Robinovitch et al., 2002; Mackey and Robinovitch, 2006; Afschrift et al., 2015), indicate that muscle strength influences the efficacy of the COP strategy. Increases in the rate of force development allow the COP to shift faster, and increases in maximal ankle plantar flexion torque allow the COP to shift further toward the edge of the BOS. Our exploratory analysis did not reveal any changes in the application of the COP strategy after RT. This is in line with our prior simulation study, which suggests that only severe muscle weakness, not present in our group of community -dwelling healthy older adults, limits the COP strategy (Afschrift et al., 2015). Hence, muscle strength might not have limited the use of COP strategies in our group of healthy older adults explaining why increases in strength after RT did not result in improved reactive balance performance. Note that ankle plantarflexion strength might be more relevant for reactive standing balance than knee-extensor strength, which was assessed in this study. We do, however, expect that RT induced similar improvements in maximal ankle plantarflexion and knee-extension torque given that the RT protocol imposed similar training demands on both muscle groups.

### Specific Adaptations Induced by PBT and RT Do Not Improve Balance in Non-trained Tasks

Improvements in reactive standing balance after PBT did not generalize to walking balance. PBT studies rarely evaluated reactive balance in tasks different from the trained task and thus little is known on generalizability of PBT. van Duijnhoven et al. (2018) used a training exercise paradigm that was similar to the one used in this study, i.e., platform translations, and found improvements in reactive balance performance in a lean-and-release task after training. Since they had no control group, it is unclear whether improvements in the lean-and-release task were due to the perturbation training or to a learning effect. Bierbaum et al. (2013) showed that reactive balance to perturbations during walking improved in a group that performed a 14-week training consisting of functional exercises of the balance-correcting mechanisms but not in a group that combined these same exercises with strength training. The evidence of this study is thus not straightforward but, but similar to our study, these results indicate again that strength should not be the main target of fall prevention but also that generalizability of such reactive balance exercises is complicated to understand. We propose that the analysis of how the different balance-correcting mechanisms are applied throughout the tested reactive balance task, is useful to provide more insight on the generalizability of training interventions.

The reduced reliance on stepping during reactive standing balance did not generalize to walking, which might suggest that different mechanisms drive the selection of a strategy during both tasks. Older adults step more in response to perturbations of standing (Pai et al., 1998; Afschrift et al., 2017) and use larger corrections in step length in response to perturbations of walking (Afschrift et al., 2019) than young adults, which suggests an age-related change in a common mechanism that determines stepping strategies during both standing and walking. Yet, the effect of PBT on stepping during perturbed standing and not during perturbed walking, questions the existence of such a common mechanism. After PBT, the step initiation threshold increased reducing step incidence (Pai et al., 1998). However, healthy older adults did not reduce step length corrections during perturbed walking after PBT, and thus relied similarly on stepping strategies walking pre- and post-intervention. This lack of generalizability might have been related to different instructions, older adults were explicitly instructed to avoid step responses during perturbed standing, whereas no instructions related to step length corrections were given during perturbed walking. Alternatively, the perturbations during perturbed walking might not have been challenging enough to detect a reduced reliance on step length corrections. Yet our protocol was based on our previous work (Afschrift et al., 2019) in which we found differences in step length corrections between young and healthy older adults for the applied forward and backward perturbations. Hence, age-related changes in sensorimotor function or motor control that were not affected by PBT or RT must be at the basis of these age-related increases in step length corrections.

Narrow beam walking performance did not increase more after PBT than after RT. The observed main effect might thus originate from learning effects or both trainings have a similar effect. The difference in training paradigms between PBT and RT would suggest a learning effect, rather than training specific improvements. The task of beam walking challenges reactive balance, but in a very different way than the unexpected perturbations applied during standing and walking. First, the perturbations during beam walking are self-induced. Second, balance is mainly challenged in the frontal plane (Sawers and Hafner, 2018b). The PBT training focused strongly on balance in the sagittal plane. We did provide medio-lateral perturbations during training, but these did not present a strong balance perturbation as standing with the feet at shoulder width is stable. The main purpose of the medio-lateral perturbations was a better randomization, making the perturbation direction more unpredictable.

The two interventions were not standardized in terms of training dosage, which is a limitation when to interpret the efficiency of the two interventions for improving reactive balance. However, the main goal was to analyze whether training specific improvements would generalize to a task that was not trained. Further, we doubt that increasing the dosage of the perturbation training intervention to a dosage similar to the resistance training might lead to better generalization to other tasks, since the achieved improvements in the trained task were already strong. The purpose of this study was not to provide equal dosages of each intervention, but rather to compare two interventions—including their typical dosages—that have previously been shown to induce specific improvements.

## Conclusion

By comparing the effects of a RT and PBT interventions we aimed to get more insight into the mechanisms through which PBT affects reactive balance. Our findings indicate that the effects of PBT are specific to the trained task, which was standing balance, and do not necessarily generalize to other modes of locomotion. An exploratory analysis suggests that reductions in step incidence following perturbations of standing were the result of changes in the sensorimotor transformation that determine the initiation of a stepping response, rather than adaptations in the application of hip and COP strategies. Muscle strength might play a role in the age-related decrease in reactive balance performance but muscle strength was not the primary factor limiting reactive balance in healthy older adults as reactive standing balance did not improve after increasing strength (RT). In contrast, reactive balance improved after PBT, which did not alter strength. For better insight in the generalizability of training different balance-correcting strategies we suggest quantifying the reliance on these separate strategies for both the trained and assessment tasks in future research.

## Data Availability Statement

The raw data supporting the conclusions of this article will be made available by the authors, without undue reservation.

## Ethics Statement

The studies involving human participants were reviewed and approved by Ethical Committee Research UZ/KU Leuven. The patients/participants provided their written informed consent to participate in this study.

## Author Contributions

TV, MA, SD, EV, KK, and FD conceived and designed research. TV, MA, and SD performed experiments. TV and MA analyzed data. TV, MA, and FD interpreted results. TV prepared figures and drafted manuscript. TV, MA, SD, EV, KK, and FD edited and revised manuscript. All authors contributed to the article and approved the submitted version.

## Funding

TV and MA were funded by FWO (Research Foundation Flanders): TV (1S82320N) and MA (12ZP120N), which allowed performing this research.

## Conflict of Interest

The authors declare that the research was conducted in the absence of any commercial or financial relationships that could be construed as a potential conflict of interest.

## Publisher's Note

All claims expressed in this article are solely those of the authors and do not necessarily represent those of their affiliated organizations, or those of the publisher, the editors and the reviewers. Any product that may be evaluated in this article, or claim that may be made by its manufacturer, is not guaranteed or endorsed by the publisher.
